# Early Resistance of Non-virulent Mycobacterial Infection in C57BL/6 Mice Is Associated With Rapid Up-Regulation of Antimicrobial Cathelicidin *Camp*

**DOI:** 10.3389/fimmu.2018.01939

**Published:** 2018-09-03

**Authors:** Lucille Adam, Moisés López-González, Albin Björk, Sandra Pålsson, Candice Poux, Marie Wahren-Herlenius, Carmen Fernández, Anna-Lena Spetz

**Affiliations:** ^1^Department of Molecular Biosciences, The Wenner-Gren Institute, Stockholm University, Stockholm, Sweden; ^2^Rheumatology Unit, Department of Medicine, Karolinska Institutet, Karolinska University Hospital, Stockholm, Sweden

**Keywords:** mycobacterial infection, BCG, innate immunity, lung infection, CRAMP, *Camp*, alveolar epithelial cells (AEC)

## Abstract

Early clearance of tuberculosis is the successful eradication of inhaled bacteria before the development of an adaptive immune response. We previously showed, by utilizing a non-virulent mycobacteria infection model, that C57BL/6 mice are more efficient than BALB/c in their control of bacterial growth in the lungs during the first weeks of the infection. Here, we assessed early (within 1–3 days) innate immune events locally in the lungs to identify factors that may contribute to the control of non-virulent mycobacterial burden. We confirmed that C57BL/6 mice are more resistant to infection compared with BALB/c after intranasal inoculation with mycobacterium. Transcriptomic analyses revealed a remarkably silent signature in C57BL/6 mice despite effective control of bacterial growth. In contrast, BALB/c mice up-regulated genes associated with neutrophil and myeloid cell chemotaxis and migration. Flow cytometry analyses corroborated the transcriptomic analyses and demonstrated influx of both neutrophil and myeloid cell populations in BALB/c mice, while these did not increase in C57BL/6 mice. We further detected increased release of TNF-α from BALB/c lung cells but limited release from C57BL/6-derived cells. However, C57BL/6 mice showed a marked early up-regulation of the *Camp* gene, encoding the cathelicidin CRAMP peptide, post-mycobacterial exposure. CRAMP (LL-37 in human) expression in the lungs was confirmed using immunofluorescence staining. Altogether, these findings show that C57BL/6 mice can clear the mycobacterial infection early and that this early control is associated with high CRAMP expression in the lungs without concomitant influx of immune cells. The role of CRAMP/LL-37 during mycobacterial infection may be relevant for novel protective strategies, and warrants further studies of human cohorts.

## Introduction

Tuberculosis (TB) is the ninth leading cause of death worldwide and the leading cause from a single infectious agent. Currently, there are an estimated 1.3 million TB deaths among HIV-negative people and an additional 374 000 deaths among HIV-positive people (1). There is an increasing number of evidence from both human and experimental animal studies suggesting that the host genetic heterogeneity affects not only the nature and/or the magnitude of immune responses to *M. tuberculosis (MTB)*, but also differential susceptibility or resistance to the disease (2). In this respect, Sobota et al. highlighted a positive correlation between tuberculosis resistance in HIV-1 infected, highly susceptible individuals living in an endemic area and a genetic variant in a regulatory region near IL12B (3). Furthermore, there are both epidemiological and genetic evidence supporting the existence of early MTB clearance (4, 5). Individuals that can clear MTB early do not develop a positive interferon-γ release assay (IGRA) or tuberculin skin test (TST). Hence, early clearance of MTB has been defined as the successful eradication of inhaled bacteria before the development of an adaptive immune response (5). It is therefore plausible that certain innate immune factors may effectively clear MTB (6). Consistent with such hypothesis, a role of innate myeloid cells in the protection against mycobacteria after Bacillus Calmette-Guérin BCG-mediated hematopoietic stem cell reprogramming was recently demonstrated (7).

Inbred mouse strains have been defined as either permissive or non-permissive to mycobacterium infection based on their capacity to control bacterial replication. Their differential susceptibility has been associated with genetic factors (8), an example of which is the natural resistance-associated macrophage protein 1 gene *Nramp1*, located on chromosome 1. Both BALB/c and C57BL/6 mice carry the *Nramp1* allele that mediates resistance to live BCG and are generally considered as resistant to mycobacterial infection, although differences exist in their degree of susceptibility to mycobacteria (9–11). By utilizing a BCG infection model, we previously showed that C57BL/6 mice are more efficient than BALB/c in their control of BCG growth in terms of bacterial burden during the first 2 weeks after infection (9). This observation was further corroborated in a murine *M. tuberculosis* infection model (12). However, if we measured the growth of BCG at later time points, when the adaptive immune responses have developed, both mouse strains displayed a similar ability to control BCG load (9). These results point to a crucial role of innate immune responses in the early control of BCG infection. However, there is a lack in our understanding of innate responses involved in the defense during the first days post-exposure.

In this present work, we therefore focused on the very early phase post-infection (p.i.) in an attempt to define innate immune events mediating control of BCG burden in the lungs of C57BL/6 and BALB/c mice. We inoculated C57BL/6 and BALB/c mice intranasally with BCG and measured the bacterial burden 1 or 3 days later. We used an extensive flow cytometry panel to quantify potential redistribution of cells infiltrating the lungs and isolated RNA from lung tissues for transcriptomic analysis. In BALB/c mice, we found a strong up-regulation of many chemokines and genes associated with chemotaxis and migration of neutrophils and myeloid cells, which was consistent with an early (day 1-day 3) influx of such cells. However, the even more resistant C57BL/6 mice showed remarkably few changes in the pulmonary transcriptome, which was in accordance with few phenotypic cell changes in their lungs. Notably, the highly resistant C57BL/6 mice instead showed a marked up-regulation of the antimicrobial cathelicidin peptide CRAMP (LL-37 in humans) locally in the lungs. The identification of innate immune defense mechanisms in resistance to mycobacterium infection may open up new avenues or suggest that old ideas for prophylactic or therapeutic approaches could be re-evaluated.

## Materials and methods

### Animals

Mouse experiments were approved by and performed in accordance with guidelines of the Stockholm North Ethical Committee on Animal Experiments, permit number N170/15. C57BL/6, and BALB/c (6–8 weeks old) female mice were purchased from Scanbur, Denmark and housed in pathogen-free conditions at the Animal Department of MBW, Stockholm University, Sweden. Mice were acclimatized for at least 1 week before use and supervised daily. The specified pathogen free condition of the facility was confirmed by continuous use of sentinel mice.

### Intranasal BCG inoculation in mice

BCG (Pasteur strain) was obtained from M. Rottenberg (Karolinska Institutet, Stockholm, Sweden) and grown in Middlebrook 7H9 liquid medium, supplemented with albumin-dextrose-catalase (ADC), 0.5% glycerol and 0.05% Tween 80 at 37°C, 5% CO_2_ to mid-log phase (OD_600_ ~1). Bacteria were resuspended in PBS with 10% glycerol, aliquoted and frozen at −80°C. The number of colony forming units (CFU) was determined by plating serial dilutions in Middlebrook 7H11 agar plates (Karolinska University Hospital, Solna, Sweden) prepared with glycerol, oleic acid-albumin-dextrose-catalase (OADC), polymyxin B, and amphotericin B. CFU were counted after 2–3 weeks incubation at 37°C with 5% of CO_2_.

For the murine infection experiments, the frozen BCG stock was thawed and kept in culture for 3 to 4 days, until it reached an early log phase (OD_600_ ~0.5). The BCG culture was washed and resuspended in PBS to the desired CFU concentration. Mice were anesthetized with isofluorane (Baxter Medical AB, Kista, Sweden) and inoculated with ~10^6^ (range: 0.4 × 10^6^ to 1.6 × 10^6^) or 10^7^ (range: 0.7 10^7^ to 1.7 10^7^) CFU through the nostrils (15 ul per nostril given in two doses). Mice were allowed to breathe the suspension naturally into the lungs.

### Collection of lungs and cell preparation

Mice were sacrificed by cervical dislocation 6, 24, 48 or 72 hours (h) after infection with BCG. Untreated control mice were analyzed in parallel. The thoracic cage was opened to expose the heart and lungs. To determine CFU counts, the left lung was removed and disrupted in PBS with 0.05% Tween80 using the GentleMACS dissociator (Miltenyi Biotec, Lund, Sweden). Serial dilutions of the left lung homogenate were plated as described above. In initial experiments, the CFU's were measured in both the left and the right lung lobes to ascertain that similar bacterial growth was reached (Figure S1A).

After collection of the left lung, systemic blood was flushed out from the right lung lobes by performing a small incision in the left heart ventricle and perfusion of the right heart ventricle with PBS until the lungs were white. The post-caval lobe was collected in RNA later (Qiagen, Sollentuna Sweden), kept for 24 h at 4°C and then stored at −20°C prior to total RNA extraction.

The superior, middle and inferior right lobes were collected in RPMI 1640 for cell extraction. Cells were extracted by transferring the right lung lobes to digestion medium containing RPMI 1640 (Thermo Fisher Scientific, Stockholm, Sweden), liberase TM 0.125 U/ml (Roche Diagnostic, Bromma, Sweden) and DNAse I grade II 0.02 mg/ml (Roche Diagnostic). The tissue was disrupted immediately with the GentleMACS, incubated 45 min at 37°C and disrupted a second time with the GentleMACS to liberate the cells. Cell suspension was filtered through a 70 μm nylon membrane. Red blood cells were lysed for 5 min with red blood cells lysis buffer (ebioscience, Thermo Fisher) washed and resuspended in PBS for direct flow cytometry staining, or resuspended in medium for *in vitro* culture.

### Flow cytometry

Single cell suspensions extracted from the lungs were stained with a live/dead-Infrared marker (Thermo Fisher) and incubated with monoclonal antibody CD16/CD32 Becton Dickinson Biosciences (BD) to block Fc receptors, before adding a cocktail of directly conjugated mAbs directed against the following surface markers; CD11b-BV711 (M1/70, 563168), Ly6G-PerCp-Cy5.5 (1A8, 560602), CD45-BB515 (30-F11, 564590), SiglecF-PE-CF594 (E50-2440, 562757), CD103-BV421 (M290, 562771), CD11c-BV510 (HL3, 562949), CD3e-BV786 (145-2C11, 564379), CD19-BV605 (1D3, 563148), CD335-BV605 (29A1.4, 560469), and Ly6C-APC (AL-21, 560595) all purchased from BD and FceR1-PE-Cy7 (MAR-1, 25-5898-82), MHC-II-AF700 (M5/114.15.2, 56-5321-82) from Thermo Fisher (ebioscience). Data acquisition was performed with the BD LSR Fortessa (BD) and Diva software, and the analysis was performed with FlowJO X software (TreeStar, Ashland, OR). In initial control experiments, the right lobes were evaluated for the frequency of contaminating peripheral blood mononuclear cells after cardiac PBS infusion by staining with an anti-CD115 mAb (T38-320, 565249, BD), which is uniformly expressed on all circulating blood monocytes (13) but not on lung monocytes (14). The frequency of CD115^+^ cells was below 0.01% among the lung cells, arguing that we had little if any blood leukocyte contamination (Figure S1B).

### Cytokine and chemokine measurements with ELISA

Half of the cells extracted from the right lungs were resupended in 1 ml of RPMI 1640, supplemented with 10% FCS, 1% Hepes, 1% L-Glutamine, 1% Penicillin-Streptomycin and then incubated at 37°C at 5% CO_2_ during 24 h without further stimulation. Supernatants were collected and quantification of MCP-1 and TNF-α were performed using ELISA from R&D Systems Europe, Abingdon, UK and Mabtech, Nacka, Sweden, respectively.

### RNA extraction

RNA was extracted from the post-caval lung lobes using the RNeasy plus mini kit (Qiagen) according to the manufacturer's recommendations with slight differences. Briefly, the whole tissue was transferred to a mTube (Miltenyi biotech) containing 500 μl of RLT buffer supplemented with 10 μl/ml of β-mercaptoethanol before disruption with the gentleMACS dissociator. The lysates were spun during 10 min at 3000 g before transfer to a gDNA eliminator column. To improve solvent elimination before RNA elution, the washing buffer was put several minutes on the column before centrifugation and a third washing step with RPE buffer was performed. The RNA concentrations obtained were around 345 ±100 ng/ul with A260/280 and A260/230 values equal to 2.07 ± 0.02 and 2.14 ±0.05, respectively.

### Nanostring analysis

The nCounter Mouse Immunology Panel (Nanostring Technologies, Seattle, WA) was used to analyse gene expression in 24 RNA samples. The panel profiles 561 genes; 547 immunology-related genes and 14 internal reference controls. According to the manufacturer's recommendation, 50 ng of total RNA for each sample was mixed with the reagents and hybridized at 65°C overnight. Samples were processed and analyzed in the automated nCounter Prep Station (Nanostring Technologies). Data were quality controlled and normalized using the nSolver Analysis Software v3.0.22 (Nanostring Technologies). A detection threshold was defined using the mean plus 3 standard deviations of the negative controls. A total of 59 genes had values under the level of detection in samples from both mouse strains at both time points and were therefore removed, leaving 488 genes for further analysis. Analysis of enriched GO terms was performed using the Gene Ontology enrichment analysis and visualization tool (GOrilla) (15, 16) using unranked target and background gene lists. Heat maps were generated using the online Morpheus software (software.broadinstitute.org/morpheus/).

### Real-time PCR

Reverse mRNA transcription was first performed using 2 μg RNA, 3.57 μM oligodeoxythymidylic acid (oligo dT), 710 μM mix 2′-deoxynucleosides 5′-triphosphates (dNTP). Reactions were heated to 65°C for 5 min followed by at least 1 min at 4°C to allow annealization to occur. A final concentration of 5 mM of Dithiothreitol (DTT), 200 U of Superscript III RT and First-Strand buffer 1X were then added to each reaction. One cycle of PCR included 5 min at 25°C, 60 min at 50°C, 15 min at 70°C, followed by the end of the cycling by unlimited time at 4°C. The amount of the cDNA obtained was measured with Nanodrop. Real-time PCR was performed with 100 ng of cDNA and a final concentration of 0.2 μM of forward and reverse primers and SYBR Green mix. Following an initial denaturation step for 10 min at 95°C, 45 cycles were run, constituted by 10 sec at 95°C, 20 sec at 50°C, and 20 sec at 72°C. The amount of double stranded PCR product was measured as SYBR green fluorescence at the end of the extension phase. All PCR reactions yielded only a single product species as revealed by melting point analysis. Primers sequences were selected according to Rivas-Santiago group (17) and the NCBI Blast primers online software from the following targets: *Camp* forward (5′-GCCGCTGATTCTTTTGACAT-3′) and *Camp* reverse (5′-AATCTTCTCCCCACCTTTGC-3′); the hypoxanthine-guanine phosphoribosyl transferase (*hprt*) forward (5′-TCCTCCTCAGACCGCTTTT-3′) and *hprt* reverse (5′-CCTGGTTCATCATCGCTAATC-3′). The relative expression of each sample was calculated using mouse cDNA *hprt* levels as a reference in all experiments and the delta-delta CT method.

### Immunofluorescence and imaging analysis

Lungs from uninfected mice, or lungs obtained 1 day after infection with 10^7^ BCG, were perfused with 1:1 OCT/PBS intra-tracheal and directly snap frozen in OCT (Fisher scientific, Stockholm, Sweden). Twelve μm tissue sections were mounted on super Frost glass slide (Fisher Scientific) and fixed for 20 min with 4% formaldehyde (Sigma-Aldrich, Stockholm, Sweden). The endogenous peroxidase was quenched with Peroxo-block (Invitrogen, ThermoFisher). Sections were blocked with PBS supplemented with 10% normal Horse serum (Invitrogen) and 0.5% Triton X-100. In separate experiments bronchoalveolar lavage (BAL) was obtained. Mice were sacrificed by cervical dislocation, whereafter the muscles around the neck were removed to expose the trachea; a cannula (BD Venflon #391452) was inserted in the trachea and injected slowly with 0.5 mL of PBS (4 × 0.5 ml in total). The PBS was slowly aspirated four times and the BAL was collected in Eppendorf tubes. Red blood cells were lysed with 1X red blood cells lysis buffer for 5 min. Cells were washed with PBS, counted, resuspended in 100 μL of PBS and cytospun onto POLYSINE slides (ThermoFisher) followed by fixation for 20 min with 4% formaldehyde (Sigma-Aldrich, Stockholm, Sweden). Lung sections or BAL cells were incubated overnight at 4°C with rabbit anti-mouse CRAMP (polyclonal, BS-4735R, Nordic Biosite, Täby, Sweden), followed by incubation with a fluorochrome-conjugated secondary antibody goat anti-rabbit-Cy3 (Jackson Immunoresearch, Ely, United Kingdom). For the detection of the surface markers CD326 (EpCAM, G8.8, 11-5791-82, Invitrogen, ThermoFisher) and F4/80 (BM8, 123107, Biolegend, London, United Kingdom) the slides were incubated with monoclonal antibodies directly conjugated with FITC for 1 h at room temperature. An irrelevant antibody was used as a negative control (anti-CD25-FITC cat no 555431, BD). Sections were mounted with Vectashield-DAPI (Vector Laboratories, purchased from BioNordika, Sweden). Six to twelve representative pictures of each condition were taken with a 20x objective using a Zeiss Axio Observer Z1 microscope. The mean fluorescence intensities were measured by using a threshold of 150 to exclude the negative, non-CRAMP expressing cells and the setting integrated density in ImageJ.

### Statistics

Data were analyzed with Prism 5.0 (graph-pad Software Inc, La Jolla, CA, USA). Differences between two groups were analyzed using the Mann Whitney test. Differences between several groups were analyzed using the two way ANOVA test with a Bonferroni post-test. Relationships between cellular frequencies and CFU counts were determined using the Spearman rank correlation after having tested our data set for normality using the Kolmogorov-Smirnov test (with Dallal-Wilkinson-Lilliefor *P*-value). We also performed unsupervised hierarchical clustering of the log2 fold change of a data set of genes expressed after BCG inoculation in mice using the one minus Pearson's correlation. Functional gene set enrichment analysis of GO terms was performed using the GOrilla tool.

## Results

### Lower BCG load in lungs of C57BL/6 mice compared to BALB/c detected one and three days after intranasal challenge

To reveal potential early mechanisms occurring after exposure to mycobacteria, we inoculated C57BL/6 and BALB/c mice with live BCG growing at the early exponential phase and analyzed responses and BCG load in the lungs 1 day (D1) or 3 days (D3) p.i.

**Figure 1:**
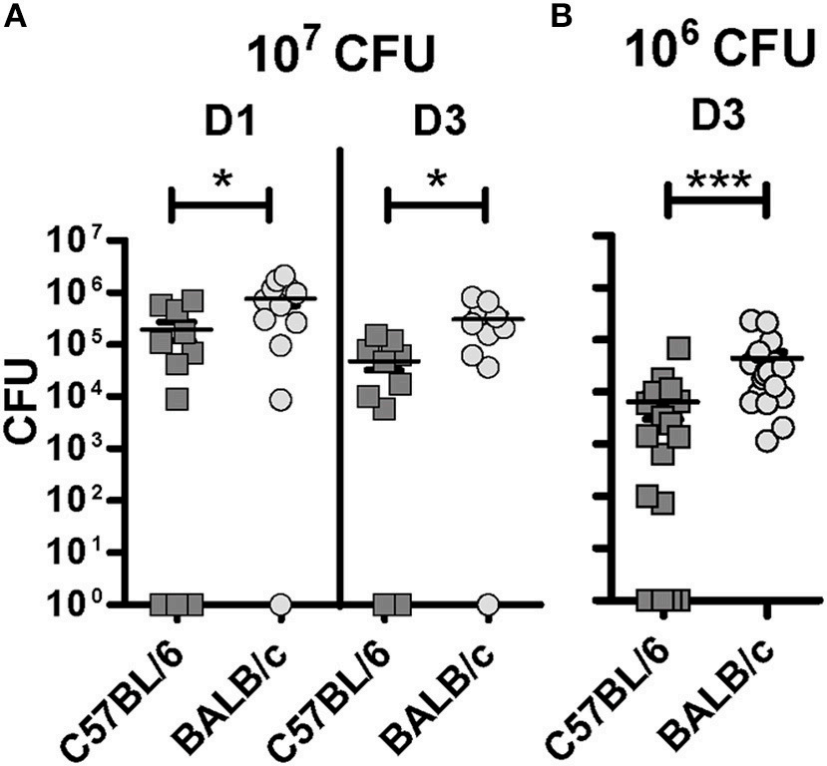
C57BL/6 show improved control of the BCG load in the lungs compared with BALB/c at early stages post-inoculation. Colony forming unit (CFU) measured from the left lung of C57BL/6 and BALB/c mice either 1 or 3 days post-inoculation with a BCG inoculum of 10^7^ CFU **(A)** or 10^6^ CFU **(B)**. Three independent experiments, with three to six mice per group, were performed at D1 (10^7^ CFU); two independent experiments, with five mice per group, were performed at D3 (10^7^ CFU); and four independent experiments, with five mice per group, were performed at D3 (10^6^ CFU). The graph shows means ± SEM. Mann Whitney test was used to compare CFU between both mouse strains, *p < 0.05, ***p < 0.001.

A significantly lower BCG burden was observed in C57BL/6 compared with BALB/c mice (Figure 1). This difference occurred already at D1 (*p* = 0.0308) and prevailed at D3 (*p* = 0.0139) p.i using 10^7^ CFU for infection (Figure 1A). In some animals the CFU numbers were as high as 2 × 10^6^, and as high infectious doses can hide differences in resistance between individuals (18, 19), we decreased the inoculum to 10^6^ CFU to enhance potential differences in susceptibility of the mouse strains. Indeed, we detected a significantly lower BCG load (*p* < 0.0001) in the lungs of C57BL/6 mice compared with BALB/c D3 p.i. using 10^6^ CFU (Figure 1B). Notably, in 7/20 of the C57BL7/6 mice, no BCG could then be isolated at D3, suggesting that these mice were very competent in the early control of mycobacterium infection. Taken together, these data support the hypothesis that innate mechanisms may be involved in the primary control of BCG infection, and that these mechanisms display differential efficacy in C57BL/6 and BALB/c mice.

### Early influx of neutrophils and CD11b^+^ APC in BALB/c mice after BCG challenge, while cell subset distribution remained unaltered in C57BL/6 mice

To increase the understanding of innate mechanisms taking place during mycobacterial infection, we established a 12-color flow cytometry panel to identify leukocyte subsets in mouse lungs (20–22) (Figure S2). Alveolar macrophages (CD11c^+^CD11b^−^; subset 1) and eosinophils (CD11c^−^CD11b^high^; subset 2) were characterized according to their expression of SiglecF. Polymorphonuclear neutrophils (subset 3) were identified through their high expression of CD11b and their exclusive expression of Ly6G. Among the CD11c^int^MHCII^+^ antigen presenting cells (APC), the CD103^+^CD11b^−^ subset corresponds to CD103^+^dendritic cells (DC; subset 4) and the CD103^−^CD11b^+^ population contains CD11b^+^DC and interstitial macrophages (subset 5). Monocytes (CD11b^+^MHCII^−^) were subdivided into Ly6C^−^CD11c^mid^ monocytes (subset 6) and Ly6C^+^CD11c^−^ monocytes (subset 7). Finally, we also identified a population of FcεR1^+^ cells corresponding to FcεR1 expressing granulocytes (subset 8).

**Figure 2:**
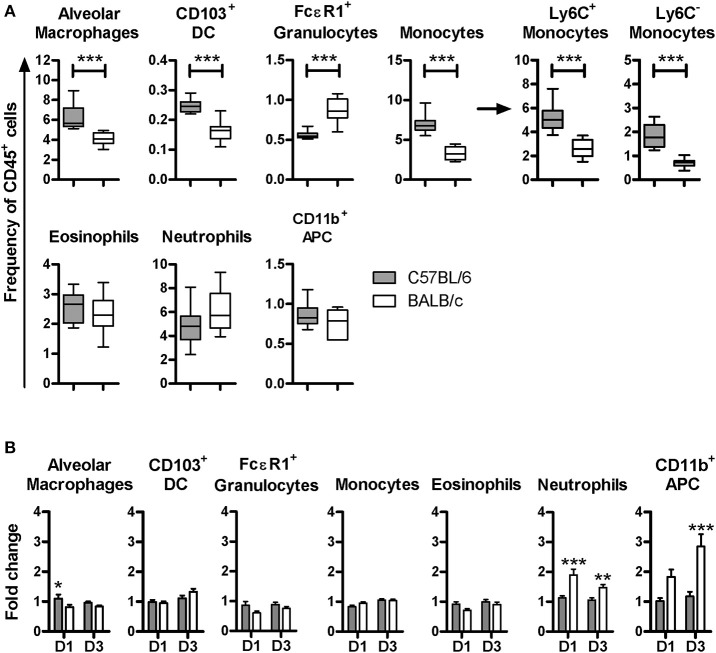
C57BL/6 and BALB/c displayed different proportions of lung cell subsets at steady state and a distinct pattern of cellular influx post-infection with BCG. Box plot representation of the frequency of each lung cell subset isolated from the right lung lobes among CD45^+^ cells (dark boxes C57BL/6 and white boxes BALB/c). Box plots show the mean, the minimal and maximal value measured. The figure corresponds to two independent experiments with five mice per group. Mann Whitney test was used to compare frequencies between the mouse strains, ***p < 0.001 **(A)**. Higher inflammatory cellular influx occurred in the lungs of BALB/c than in the lungs of C57BL/6 mice. Single cell suspensions were obtained from superior, middle and inferior right lung lobes. Half of these cells were used for cytometry analysis to determine the cellular frequency in uninfected mice, or at D1 and D3 post-BCG inoculation with 10^7^ CFU. Results are expressed as fold change. The frequencies of lung subsets after infection were divided by the average in uninfected individuals. Data were expressed as the mean ± SEM and correspond to three independent experiments, with three to six animals per group at D1, four independent experiment with five animals per group at D3. The average of lung subset frequencies in uninfected animals were calculated using data from 20 animals from five independent experiments **(B)**.

Although the uninfected C57BL/6 and BALB/c mouse strains displayed similar lung cell subsets (Figure S3), their relative frequencies were different. In particular, uninfected C57BL/6 mice had higher frequencies of alveolar macrophages, Ly6C^−^ and Ly6C^+^ monocytes as well as CD103^+^DC. In contrast, they displayed a lower frequency of FcεR1^+^ granulocytes. CD11b^+^APC, neutrophils and eosinophils were equally represented in both mouse strains (Figure 2A). These data show that C57BL/6 and BALB/c mice feature differential proportions of leukocytes in the lungs, suggesting that mycobacterium may encounter a different microenvironment upon infection.

To evaluate potential early phenotypic changes in the subsets present in lungs after BCG inoculation, we performed flow cytometry analyses at D1 and D3 p.i. with 10^7^ CFU. We observed significant differences in the cellular influx between BALB/c and C57BL/6 mice. The recruitment of neutrophils and CD11b^+^APC was significantly higher in BALB/c compared with C57BL/6. However, these populations remained almost unchanged in the lungs of C57BL/6 p.i. (Figure 2B). The frequencies of populations such as eosinophils, CD103^+^DC, alveolar macrophages and monocytes, remained similar to the uninfected mice with a fold change around 1 in both mouse strains.

**Figure 3:**
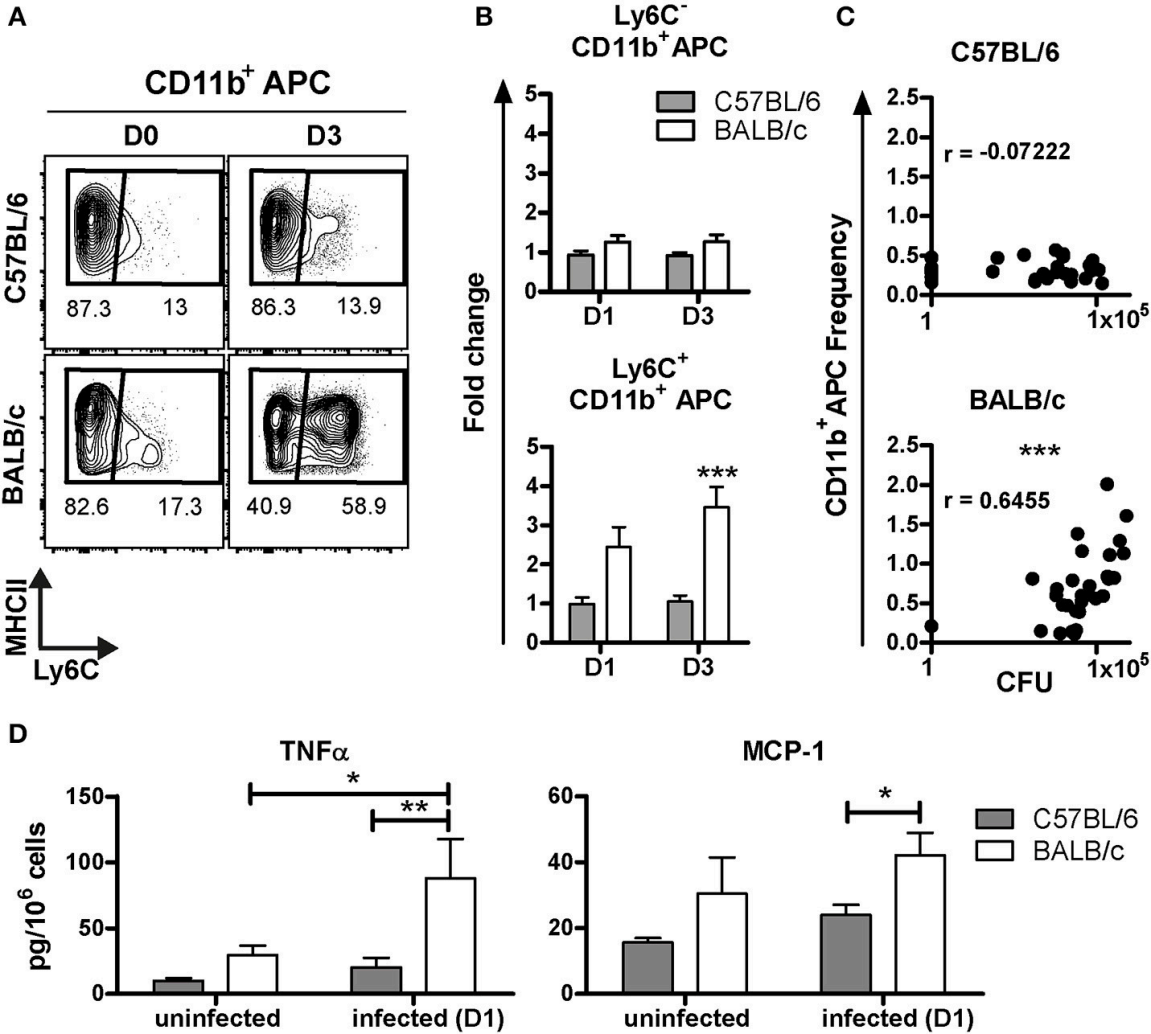
Early recruitment of inflammatory CD11b^+^APC in BALB/c but not in C57BL/6 mice post-BCG challenge. Contour plot representation of Ly6C and MHC class II expression among the CD11b^+^APC subset in C57BL/6 and BALB/c. Each plot correspond to one representative animal from an uninfected mouse, or at D3 post-inoculation with 10^7^ CFU **(A)**. Changes in the frequency of CD11b^+^APC, positive or negative for Ly6C, at D1 or D3 p.i. with 10^7^ CFU, were depicted as fold change. The frequencies of Ly6C^+^/Ly6C^−^ CD11b^+^APC after infection were divided by the average of the frequencies measured in uninfected individuals. Data are expressed as Mean ± SEM and correspond to one experiment with six animals per group at D1 and two independent experiments with five animals per group at D3. The average frequencies in uninfected animals were calculated with 12 animals from four independent experiments. Two way ANOVA with Bonferroni post-test was used to compare C57BL/6 and BALB/c mice, ***p < 0.001 **(B)**. XY graph representation of the frequencies of CD11b^+^Ly6C^+^APC subset measured in the right lung lobes and the CFU count measured in the left lung for each mouse D3 p.i. with 10^6^ or 10^7^ CFU, *n* = 30. CFU and frequencies values were tested for normality with Kolmogorov-Smirnov test (with Dallal-Wilkinson-Lilliefor *P*-value). CFU but not the frequencies values passed the normality test. Consequently, the nonparametric two-tailed Spearman correlation test was used to determine the degrees of correlation between CD11b^+^Ly6C^+^APC frequencies and CFU count. The Spearman *r* coefficient is indicated on the graph, ***p < 0.0001 **(C)**. Soluble factors were measured by ELISA using supernatants obtained from unsorted lung cells extracted directly *ex vivo* (without further stimulation) after isolation from right lungs at D1 p.i. To allow the cells to release soluble factors into the medium, cells were directly re-suspended in 1ml of cell culture medium and incubated for 24 h at 37°C. Data were calculated per million of cells and shown as the mean ± SEM from three to four mice per group for uninfected group (two independent experiments) and six mice per group at D1 post-inoculation (one experiment). Nonparametric Mann Whitney test was used to compare cytokine secretion between both mouse strains, *p < 0.05, **p < 0.01 **(D)**.

It is well established that recruitment of inflammatory APC expressing Ly6C, derived from Ly6C^+^ monocytes, can occur upon inflammation or infection (23–25). These inflammatory APC were shown to be essential in pathogen growth control, and direct killing in the context of HSV-2 and *L. monocytogenes*, respectively (24, 25). To assess the presence of inflammatory APC after BCG inoculation, the expression level of Ly6C among the CD11b^+^APC was studied. Two subpopulations were distinguishable; the CD11b^+^Ly6C^−^ and CD11b^+^Ly6C^+^ subsets (Figure 3A). At D1 and D3 p.i., the frequency of the CD11b^+^Ly6C^−^ subset remained unchanged and was similar in both mouse strains, as compared to the frequency measured in uninfected mice (Figure 3B). However, we detected a significant increase of the CD11b^+^Ly6C^+^ subset in BALB/c, with a 2.4 ± 0.5, and 3.5 ± 0.5 fold increase at D1 and D3, respectively. Notably, we did not observe any change in this subset before and after infection in C57BL/6 mice.

We observed a positive correlation between the frequency of CD11b^+^Ly6C^+^ APC with the CFU count in BALB/c (*p* = 0.0001) but not in C57BL/6 (*p* = 0.7045; Figure 3C). These data suggest that the quantity of BCG in the lungs influenced the magnitude of recruitment of inflammatory CD11b^+^APC in BALB/c, while the C57BL/6 mice did not mount an extensive recruitment of CD11b^+^APC. The large difference in CFU between C57BL/6 and BALB/c mice can be appreciated in Figure 3C.

We then quantified cytokines released from lung cells during 24 h cultures directly *ex-vivo* without further stimulation. These experiments were performed to validate cytokine protein expression and enable comparison with results obtained with transcriptomic analyses (next section), therefore we attempted to measure proteins in the supernatant released directly *ex-vivo* without further stimulation. It is conceivable that relatively low levels of protein can be measured using this approach as compared with *in vitro* re-stimulation protocols. We were able to detect significantly increased release of TNF-α from BALB/c lung cells extracted at D1 p.i. with BCG, as compared with uninfected BALB/c mice. However, C57BL/6 mice did not respond with increased TNF-α production after BCG infection. We found that BALB/c mice released significantly higher amounts of MCP-1 compared with C57BL/6 following BCG exposure (Figure 3D), which is in line with increased influx of monocytes in BALB/c mice. However, there was only a trend of increased release of MCP-1 comparing BALB/c mice D0 and D1 post-infection.

Altogether, these results show that the C57BL/6 mice harbored higher frequencies of myeloid cells in the lungs prior to infection and, neither recruited inflammatory cell populations, nor produced high level of inflammatory cytokines such as TNF-α or MCP-1 at early stages (D1) post-infection but, nevertheless, showed early control of BCG load in the lungs. However, BALB/c mice exposed to BCG recruited, in particular, CD11b^+^Ly6C^+^ APC and neutrophils to the lungs.

### C57BL/6 but not BALB/c mice show rapid up-regulation of antimicrobial cathelicidin *Camp* post-BCG challenge

**Figure 4:**
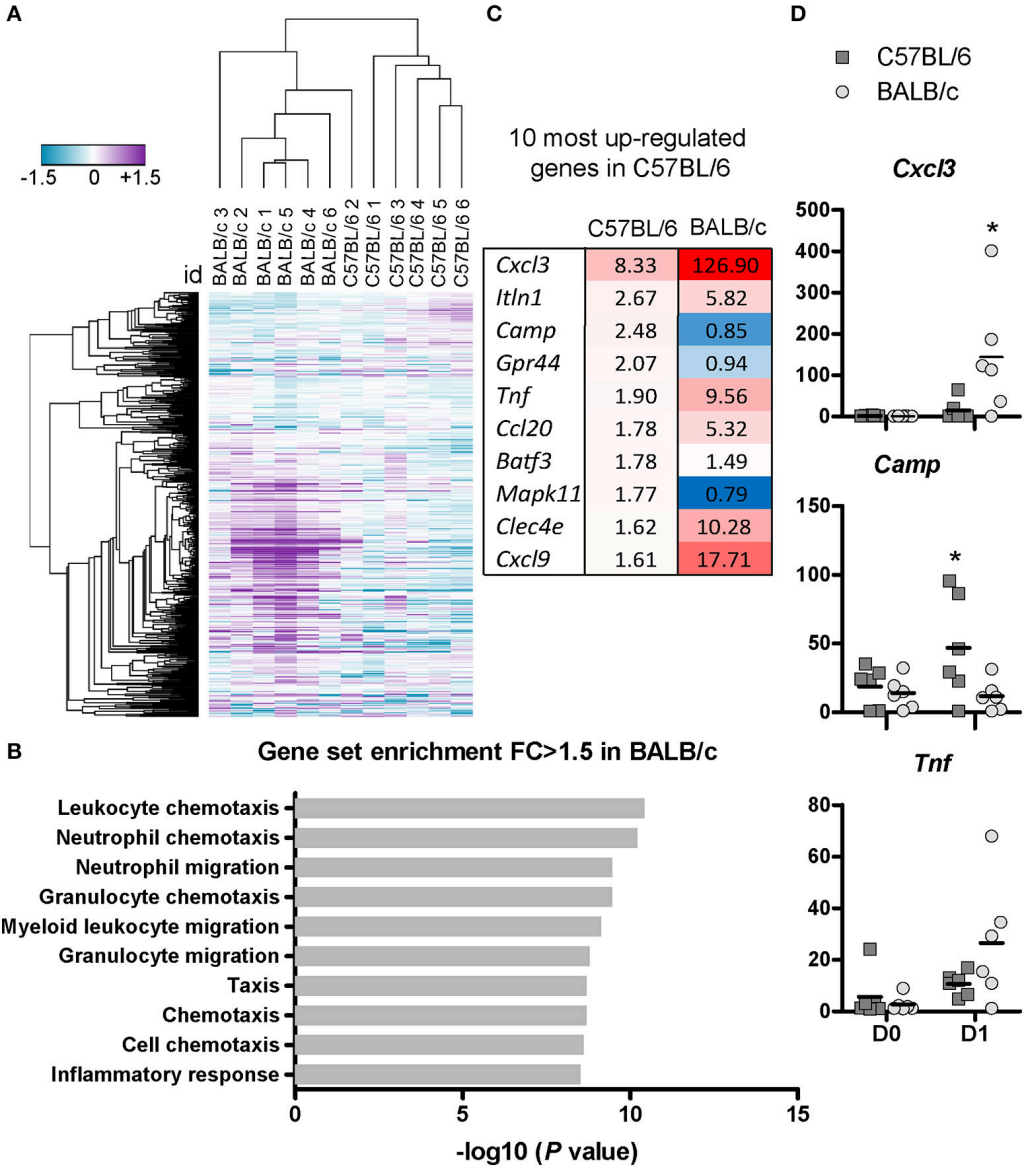
C57BL/6 and BALB/c exhibit differential gene regulation at the early stage post-BCG inoculation.Total RNA from the posterior caval right lung were extracted from uninfected mice or at D1 post-inoculation with 10^7^ CFU, and analyzed using Nanostring technology. The nCounter Mouse Immunology Panel analyzing 547 genes related to immune responses was used. Four hundred and eighty-eight genes were considered positively expressed (> negative controls mean + 3 *SD*) and used for further analyses. The heat map shows the log2 fold change calculated from the probe counts for each individual mouse at D1 divided by the average probe count of the uninfected mice of each strain (*n* = 6). Unsupervised hierarchal clustering was performed using one minus Pearson's correlation. **(A)** Functional gene set enrichment analysis of GO terms was performed using the GOrilla tool. Top 10 most enriched gene sets are displayed with their corresponding –log10 (*P*-value) **(B)**. Top 10 of the most upregulated genes in C57BL/6 mice with the corresponding level of regulation is shown also for BALB/c mice. The data are represented as fold changes with the exact fold changes indicated with the number and complemented with a color code. The increased redness indicates upregulated genes and blue color indicates downregulated genes. *N* = 6 for each mouse strains at D1 and *n* = 6 for each mouse strains at D0 **(C)**. The actual probe counts illustrate the amount of transcript synthetized in uninfected mice, or at D1 post-BCG inoculation, in both mouse strains **(D)**.

To assess the immune transcriptome signatures following mycobacterium infection, we extracted total RNA from the lungs collected at D0 and D1 post-inoculation with 10^7^ CFU and performed gene expression analysis using the nCounter Mouse Immunology Panel from Nanostring. The heat map in Figure 4A shows unsupervised hierarchical clustering illustrating the differential gene regulation after BCG exposure in C57BL/6 and BALB/c mice. The clustering is based on one minus Pearson's correlation of log2 transformed fold change values calculated as D1 gene expression in each mouse, divided by the average expression level at D0 (Figure 4A). From the utilized unsupervised hierarchical clustering, it was evident that C57BL/6 and BALB/c mice largely formed separate clusters and that there was a remarkably silent gene signature in C57BL/6 mice.

We found that 94 genes were upregulated with a fold change above 1.5 in BALB/c, while only 19 genes were upregulated in C57BL/6. Two genes were downregulated according to the same criteria in BALB/c, while 22 were downregulated in C57BL/6 (Tables S1A,B). Functional gene set enrichment analysis for genes with fold changes above 1.5 showed that BALB/c mice displayed increased expression of genes involved in chemotaxis and migration of neutrophils and myeloid cells (Figure 4B), which is in accordance with the flow cytometry data (Figure 2B). However, no gene sets were significantly enriched for C57BL/6 mice demonstrating a relatively silent, yet effective resistance to BCG.

Overall, there was a remarkably limited change of the transcriptome in C57BL/6 mice upon BCG challenge (Table S1B), and we chose to further study the ten most upregulated genes in the lungs from infected C57BL/6 mice in order to identify potential factors that could contribute to the early resistance to BCG. The fold changes of these genes measured in BALB/c are shown for comparison (Figure 4C). Among the most upregulated genes in C57BL/6 were *Cxcl3, Ccl20*, and *Cxcl9*. These genes were also upregulated in BALB/c, with an even higher fold increase. This up-regulation of chemokines in BALB/c mice is consistent with the flow cytometry data showing influx of both APC and neutrophils. The expression levels of TNF-α increased more in BALB/c than in C57BL/6, which is in accordance with the ELISA data (Figure 3D). The direct probe counts illustrate the degree of heterogeneity in each group, with some mice responding strongly post-infection while some appeared to be poor responders even though they were challenged with the same inoculum (Figure 4D).

**Figure 5:**
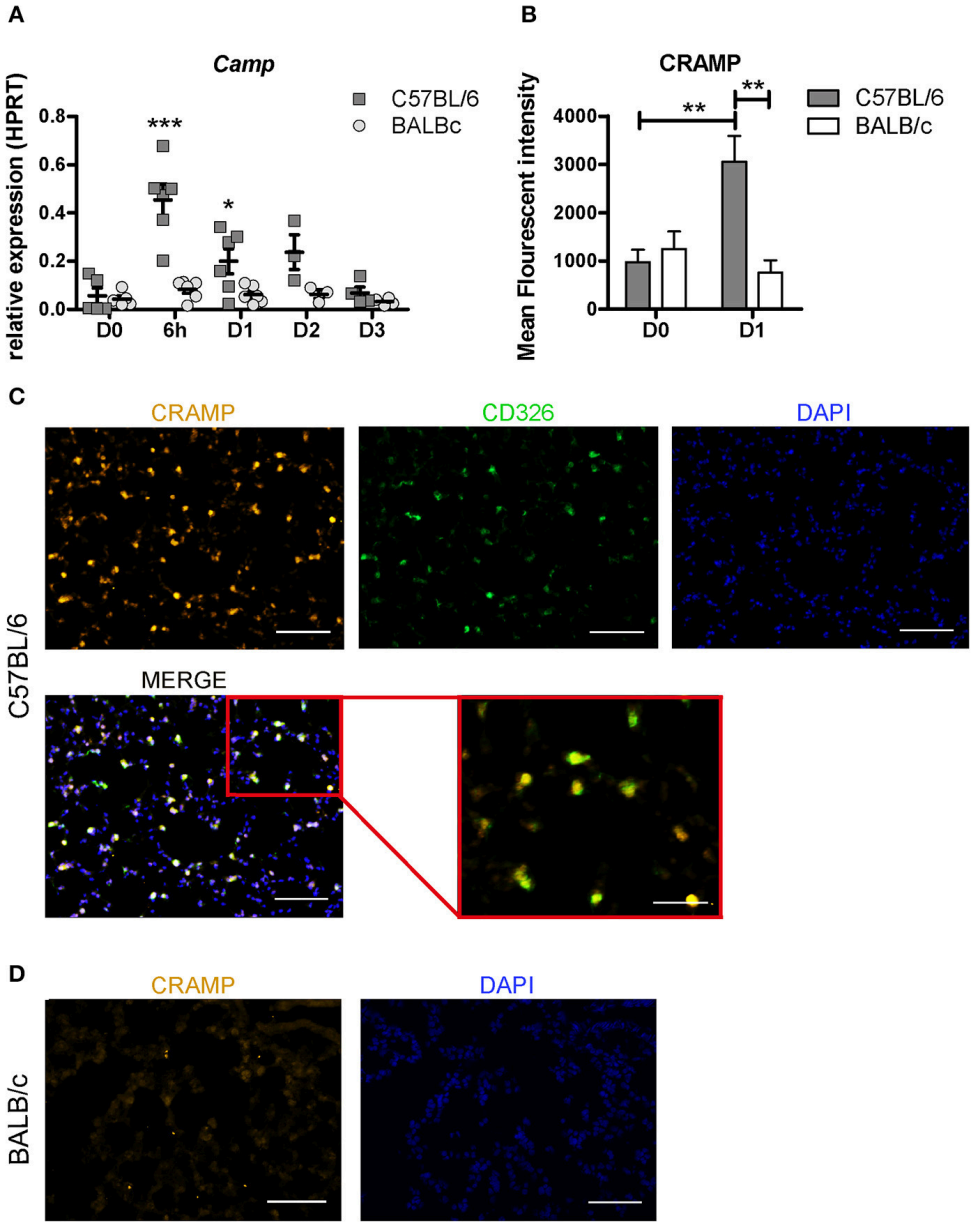
C57BL/6 mice show rapid up-regulation of antimicrobial cathelicidin *Camp* post-BCG challenge. The expression of the *Camp* gene was measured at 6h, D1, D2, and D3 post-infection with 10^7^ BCG CFU in C57BL/6 and BALB/c mice using real time qPCR. Relative quantification of *Camp* was related to the housekeeping gene HPRT. Two way ANOVA with Bonferroni Post-test was used to measure the significance in *Camp* regulation change between the two mouse strains, *p < 0.05; **p < 0.001, D0, *n* = 5; D1, *n* = 6; D2, *n* = 3; D3, *n* = 4. **(A)** Immunofluorescence analyses were performed on lung sections and quantified by measuring the Mean Fluorescent intensity (MFI) of CRAMP stained tissue at D1 p.i. and in uninfected animals by a blinded observer. The graph depicts the MFI of 12 representative pictures for each condition. A threshold of 150 pixels was used to exclude non-CRAMP positive cells, resulting in a non-detectable isotype control staining. The MFI was determined using integrated density in ImageJ. Mann Witney test was used to measure the significance of the difference in MFI comparing the conditions and the different time-points within the conditions, ***p < 0.001 **(B)**. Co-localization of CRAMP and CD326 in lung sections of BCG exposed C57BL/6 mice. CD326 is used as a marker for epithelial cells (green), CRAMP (orange), and DAPI (blue). Data shown are representative for D1 p.i. **(C)** Representative CRAMP (orange) and DAPI (blue) staining of a BALB/c lung section D1 p.i. **(D)**.

We found that among the top 10 most up-regulated genes in C57BL/6 was *Camp*, encoding the cathelicidin antimicrobial peptide CRAMP (Figure 4D). The CRAMP peptide features direct killing properties effective against various bacterial strains including mycobacterium (26, 27). Using real-time PCR, we measured the relative expression of *Camp* over time in both mouse strains post-inoculation with 10^7^ CFU (Figure 5A). We observed a significant up-regulation of this gene in C57BL/6 at 6 h, and D1 p.i compared with the uninfected mice, with a strong peak of up-regulation at 6 h (*p* < 0.001), followed by a successive decline. However, in BALB/c the expression of *Camp* after BCG infection remained similar to the level measured in uninfected mice at all the time points studied (Figure 5A). We further evaluated expression of the encoded protein CRAMP by using fluorescence microscopy and quantification by imaging analyses, which confirmed high protein expression in C57BL/6 in a characteristic punctate staining pattern (Figures 5B,C). C57BL/6 mice responded with an increased expression of CRAMP upon BCG exposure, while we could not measure any increased CRAMP expression in BALB/c mice (Figures 5B,D). Double staining with the epithelial marker CD326 revealed that some of the CRAMP expressing cells were positive for CD326 (Figure 5C). We isolated cells from bronchoalveolar lavage (BAL) and found a high frequency of CRAMP expressing cells 1 day after BCG exposure in C57BL/6 mice. Double staining with the phenotypic macrophage marker F4/80 revealed that some of the F4/80 positive cells were expressing CRAMP (Figure S4). These results show that C57BL/6 mice were more efficient in the induction of antimicrobial peptide CRAMP following mycobacterial infection than BALB/c mice, suggesting that C57BL/6 mice possess an innate mechanism that may facilitate direct killing and early clearance of BCG.

Altogether, these observations show that different innate mechanisms were induced in response to mycobacterium infection in these two mouse strains and that the intensity of the cellular influx and inflammatory responses were not correlated with an enhanced early control of the BCG load in the lungs. Instead, the very rapid control of BCG infection that occurred in C57BL/6 mice was associated with up-regulation of the antimicrobial cathelicidin peptide CRAMP.

## Discussion

A limited number of individuals living in endemic MTB areas and in close contact with MTB patients not only do not develop the disease, but they even remain negative to various TB diagnostic tests suggesting a strong protective activity of the innate immune system operating before onset of adaptive immune responses (5). Understanding the conditions contributing to this early clearance of pathogenic agents may open up new avenues to develop novel prophylactic or therapeutic treatments. The factors contribution to resistance and susceptibility to infection is likely to be complex and the immune response multifaceted.

To gain further knowledge, we studied the very early local immune signatures in the lungs of the two mouse strains C57BL/6 and BALB/c, with differential ability to resist mycobacterial infection. The BCG vaccine is a safe live attenuated vaccine that has, and still is, widely used. In the present work, we did not use the vaccine. Instead, we used BCG growing in the early exponential phase and infected the mice primarily with live bacteria, which is very different from inoculation with the vaccine. Mice were challenged i.n. with BCG growing in the exponential phase of growth using two doses, 10^7^ and 10^6^ CFU per mouse. We measured bacterial growth in the lungs 1 and 3 days post-inoculation and confirmed our previous data showing that C57BL/6 were more efficient than BALB/c mice in the control of BCG burden. Notably, at the lower dose used, the difference between the two mouse strains was even clearer. The infection was below the detection limit in a high proportion of the C57BL/6 mice, demonstrating early efficient resistance to mycobacterium infection in these mice. The reason for this early resistance in C57BL/6 mice is likely to be complex and factors such as numbers of bacteria reaching the lungs, differential kinetics in the uptake of bacteria, different efficiency in the defense in the upper respiratory tract and epithelial lining as well as infection in different lung cell types with differential capacity to allow BCG replication, can be considered. We indeed found different frequencies of alveolar macrophages, neutrophils and DCs in the two strains of mice. These phagocytic innate cells with risk of being infected may react to the bacterium in different ways in terms of intracellular BCG replication, capability to kill the bacterium, APC functions, and BCG dissemination (28). Previous reports have demonstrated that both pulmonary macrophages (PuM) and the alveolar epithelial cells type II (AECII) were able to internalize BCG. In addition, previous work showed higher ability of internalizing BCG in PuM as compared with AECII cells (29). We here report that C57BL/6 mice displayed higher frequencies of alveolar macrophages, which are one of the major target cells for MTB and BCG. Nevertheless, these mice controlled the infection better at early time points. A recent study showed that viable neutrophils participate in the control of mycobacterium while necrotic neutrophils exerted opposite effects (30), exemplifying the complex cellular interactions occurring during TB infection that warrants further investigation.

Surprisingly and in contrast to our previous observations, we could not confirm any increase of CD11b^+^ cellular infiltrates in the lungs of C57BL/6 mice when measured D1 and D3. We here found higher levels of infiltrating neutrophils and inflammatory CD11b^+^APC in BALB/c mice, compared with C57BL/6 mice. Additionally, lower secretions of pro-inflammatory cytokines, TNFα and MCP-1, were measured *ex-vivo* from C57BL/6 lung cells 1 day post-inoculation, as compared with BALB/c lung cells. We do not have a good explanation for the difference between our previous and the current work. The major difference between the two protocols is the BCG inoculum used to challenge the mice. In the present study, we used BCG growing at the exponential phase and the mice were infected primarily with live bacteria, while in our previous work, we used bacteria from frozen stocks where the cargo was approximately 10% live and 90% dead bacteria. The use of frozen stocks for challenges are extensively used for infections, both with BCG and MTB. However, the balance between infection due to live bacteria and immune response related to dead bacteria may influence the immune response generated and should perhaps be taken into consideration in challenge experiments. Another factor to consider is the possibility of an unknown subliminal infection.

Other groups following protocols with some differences in the infection model and the time points under study have also noted strong cellular influx in the lungs of BALB/c mice, similar to the results reported here. In one of such studies, Wakeham et al. showed that the level of pro-inflammatory cytokines such as TNF-α, INF-γ, MCP-1, and IL-12, as well as influx of neutrophils in the BAL were higher in BALB/c, than in C57BL/6, during the first 14 days following the infection. The authors demonstrated a delayed immune response in C57BL/6 mice compared with BALB/c mice and it was after 27 days that the level of cytokines and cellular influx became dramatically higher in C57BL/6 than in BALB/c mice. Leepiyasakulchai et al. also studied the early stages of the infection in BALB/c and C57BL/6 mice after MTB infection (12). They similarly observed that the cellular influx was stronger in BALB/c compared with C57BL/6, in agreement with the findings reported here.

These data suggest that the recruitment of inflammatory cell populations and the induction of a stronger pro-inflammatory microenvironment may be more an indication of higher infection rates than a correlation of rapid clearance. Therefore, other factors may be responsible for the very strong control of bacterial growth in the lungs of C57BL/6 mice. It is conceivable that the differential distribution of leukocyte subpopulations in the lungs of C57BL/6 and BALB/c mice, may have contributed to the earlier control of BCG in C57BL/6 mice. To get further insights to rapidly regulated factors upon BCG exposure, we conducted immune transcriptome analysis from the lungs of C57BL/6 and BALB/c mice before and after infection with BCG. We observed a remarkably silent gene signature in C57BL/6 mice despite the early control of BCG. One of the more interesting and clear observations was the up-regulation of the *Camp* gene very early in C57BL/6 but not in BALB/c lungs. We measured a peak mRNA expression of *Camp* at 6h with a decline and return to base line levels by D3. By using RT-PCR and immunofluorescent staining of the lungs, we confirmed increased expression of *Camp* and CRAMP in C57BL/6 mice. We detected CRAMP expression among CD326 positive cells, suggesting expression in epithelial cells upon non-virulent mycobacteria challenge in C57BL/6 mice. In addition, high frequency of cells in the BAL expressed CRAMP, including F4/80 positive macrophages. The kinetic mRNA analyses showed that *Camp* expression was contracted already at D3 in C57BL/6 mice suggesting that the activities of CRAMP were tightly regulated. The rapid contraction of *Camp* expression also points toward the multifaceted responses occurring after BCG infection to obtain efficient defenses of which CRAMP might be one worthwhile exploring further.

The *Camp* gene encodes a cathelicidin antimicrobial peptide, termed LL-37 in humans and CRAMP in mice, which are amphipathic α-helical peptides that bind to bacterial outer membranes causing disruption of the bacterial cell wall (31). Increased cathelicidin expression was demonstrated to result in enhanced intracellular killing of BCG and MTB (26, 32, 33). Cathelicidins are expressed by non-myeloid cells such as epithelial cells and in neutrophils as well as macrophages (27, 32, 34). We here demonstrate that many of the epithelial cells in the lung tissues of the analyzed animals also express CRAMP. These cells were positive for CD326, the epithelial cell marker present in both alveolar epithelial cells type I (AECI) and type II (AECII). The finding of CRAMP expression in epithelial cells is in line with our previous data demonstrating the activity of AECII in macrophage activation, increasing phagocytosis and intracellular mycobacterial killing (29, 35, 36). Previous studies has suggested that LL-37 have stronger anti-microbial activity in the upper airways than in the lungs, because of the inhibitory action of surfactant, which is primarily present in the lungs and not in the upper respiratory tract (37). It also has been shown that *Camp* knockout mice were more susceptible to bacterial infections (38–40), supporting a plausible functional effect of CRAMP in C57BL/6 mice.

Cathelicidins also correlate with various aspects of protection against TB. It was reported that sunlight induces vitamin D production and that vitamin D (3) enhances the synthesis of cathelicidins. It was shown that MTB can interact with TLR2 expressed on the cell surface of macrophages leading to up-regulation of the vitamin D receptor and induction of cathelicidin (33). Interestingly, before the availability of antibiotics, exposure to sunlight and vitamin D supplementation were the methods of choice for TB treatment. Moreover, vitamin D deficiencies have been associated with development of TB disease (41). Vitamin D (3) was shown to enhance macrophage phagocytosis of MTB and increases the production of antimicrobial peptide cathelicidin and killing of MTB (41). The finding of increased CRAMP expression following challenge with BCG in the resistant C57BL/6 mice therefore warrants further investigation in humans that appear to be refractory to MTB infection.

Altogether, these data show that C57BL/6 and BALB/c mice exhibit distinct innate mechanisms in response to mycobacterial infection. BCG infection induces a stronger inflammatory response in BALB/c than in C57BL/6, with higher induction of TNF-α and an increased recruitment of inflammatory populations such as neutrophils and inflammatory CD11b^+^APC. In contrast, BCG infection induced a weak inflammatory signature in the lungs of C57BL/6 mice. However, C57BL/6 mice showed a strong up-regulation of *Camp*, encoding for the antimicrobial peptide CRAMP, displaying direct mycobacterial killing ability, with a peak of transcription 6 h post-infection, while BALB/c showed no change in the expression of *Camp*. These results suggest that C57BL/6 mice exhibit higher ability to directly kill BCG than BALB/c without the necessity of a strong inflammatory response.

## Author contributions

CF and A-LS conceived the project. LA, ML-G, CP, CF, and A-LS designed the experiments. LA and CP set up the lung cells extraction method and the flow cytometric panel of these cells. LA realized and analyzed the CFU count in the lungs of the animals, the flow cytometry, the ELISA and the RT-PCR experiments. LA, AB, and MW-H realized and interpreted the Nanostring experiments. ML-G realized the immunofluorescence lungs staining and SP performed the quantification analysis of these stainings. LA wrote the manuscript with substantial input from CF and A-LS. All the authors approved the final version of the manuscript.

### Conflict of interest statement

The authors declare that the research was conducted in the absence of any commercial or financial relationships that could be construed as a potential conflict of interest.

## Abbreviations

TB: Tuberculosis
MTB: Mycobacterium tuberculosis
IGRA: Interferon-γ releasing assay
TST: tuberculin skin test
BCG: bacillus Calmette-Guérin
p.i.: post infection
CFU: colony forming unit
BAL: bronchoalveolar lavage
APC: antigen presenting cells
DC: dendritic cells
AEC: alveolar epithelial cell
PuM: pulmonary macrophages.
