# Supplementary figures and images for: Early Resistance of Non-virulent Mycobacterial Infection in C57BL/6 Mice Is Associated With Rapid Up-Regulation of Antimicrobial Cathelicidin Camp
Source: Front Immunol. 2018 Sep 3;9:1939. doi: 10.3389/fimmu.2018.01939 (PMC6129578; doi:10.3389/fimmu.2018.01939)

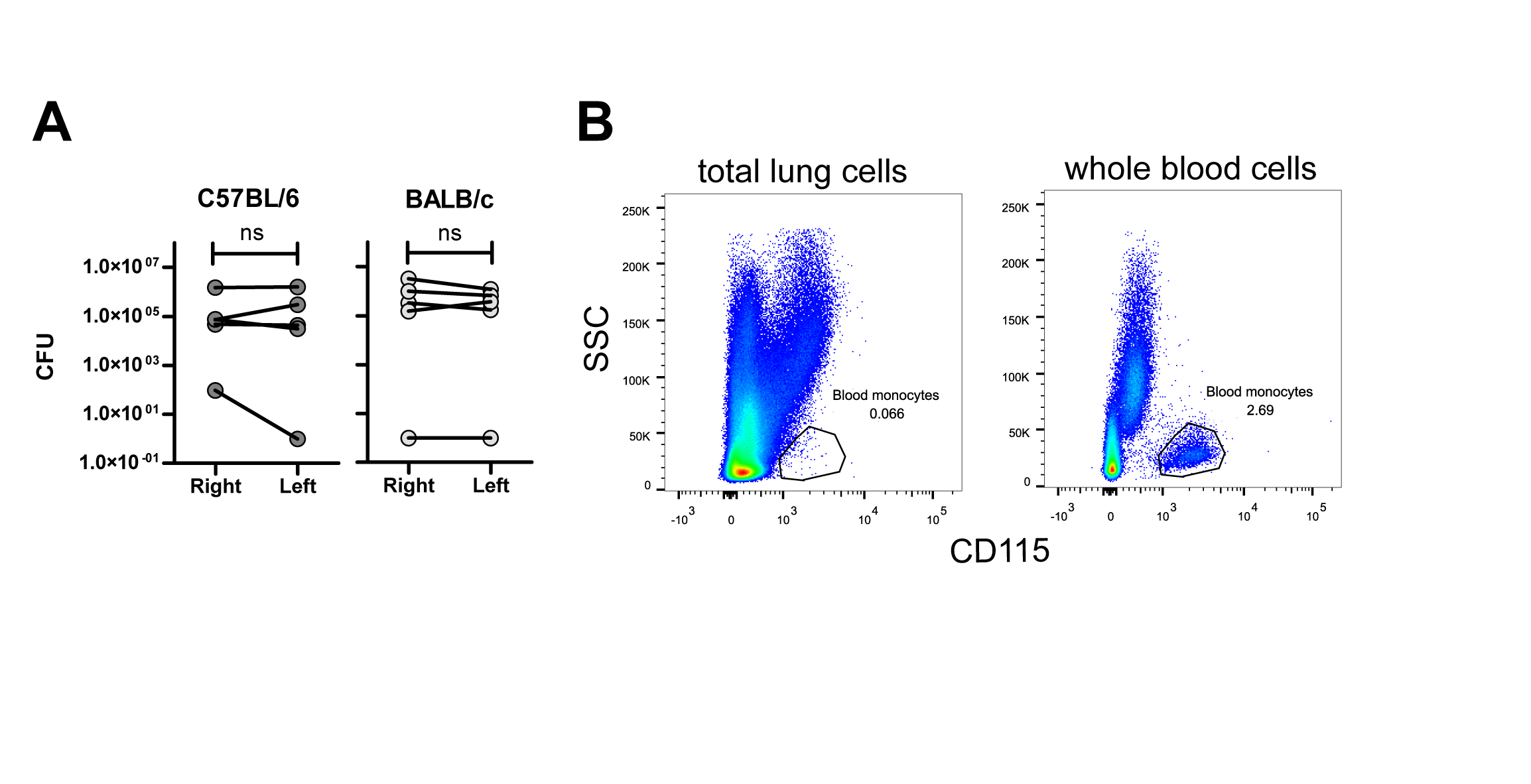

Supplement: Supplementary file 1 [file Image_1.TIF]

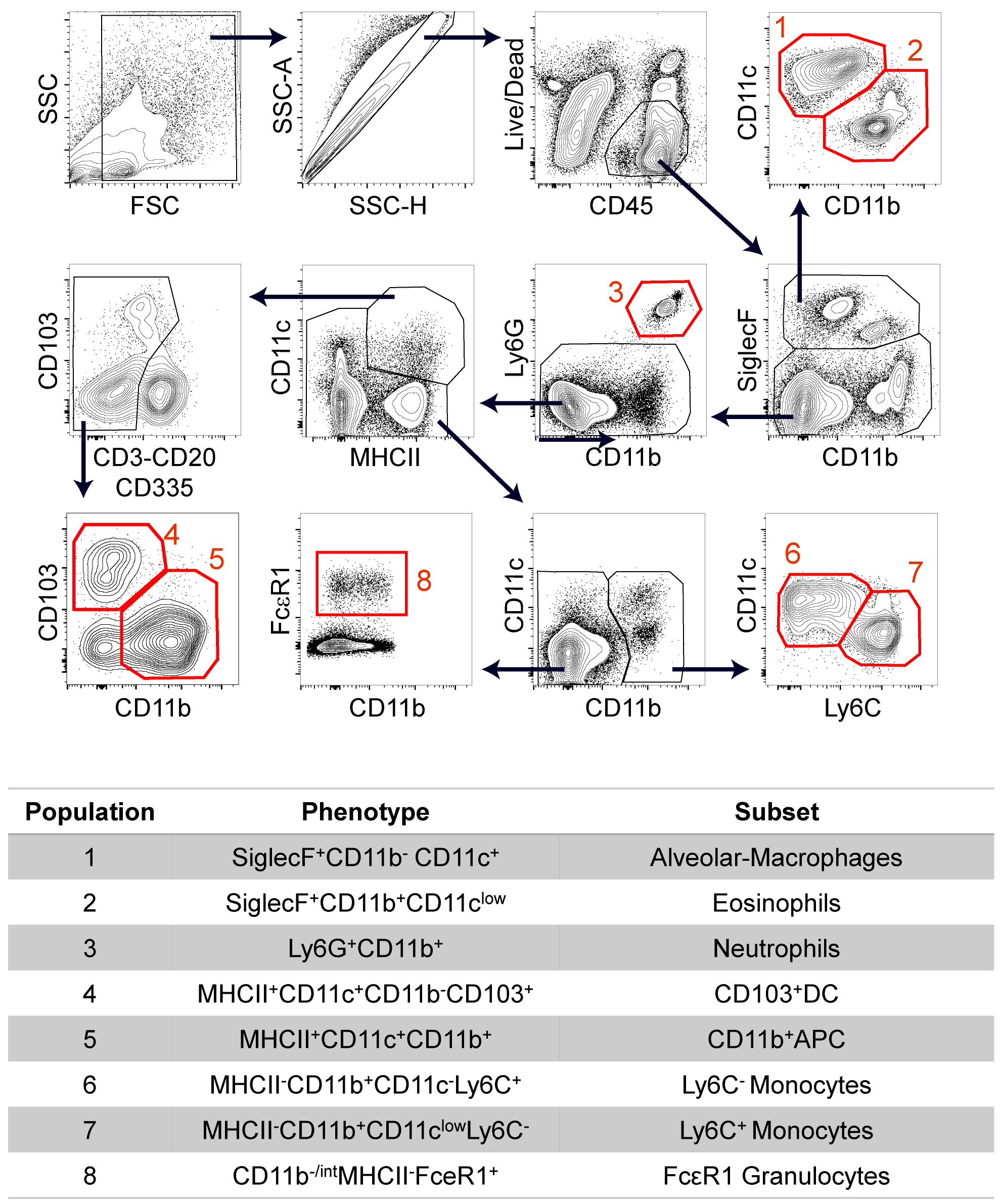

Supplement: Supplementary file 2 [file Image_2.JPEG]

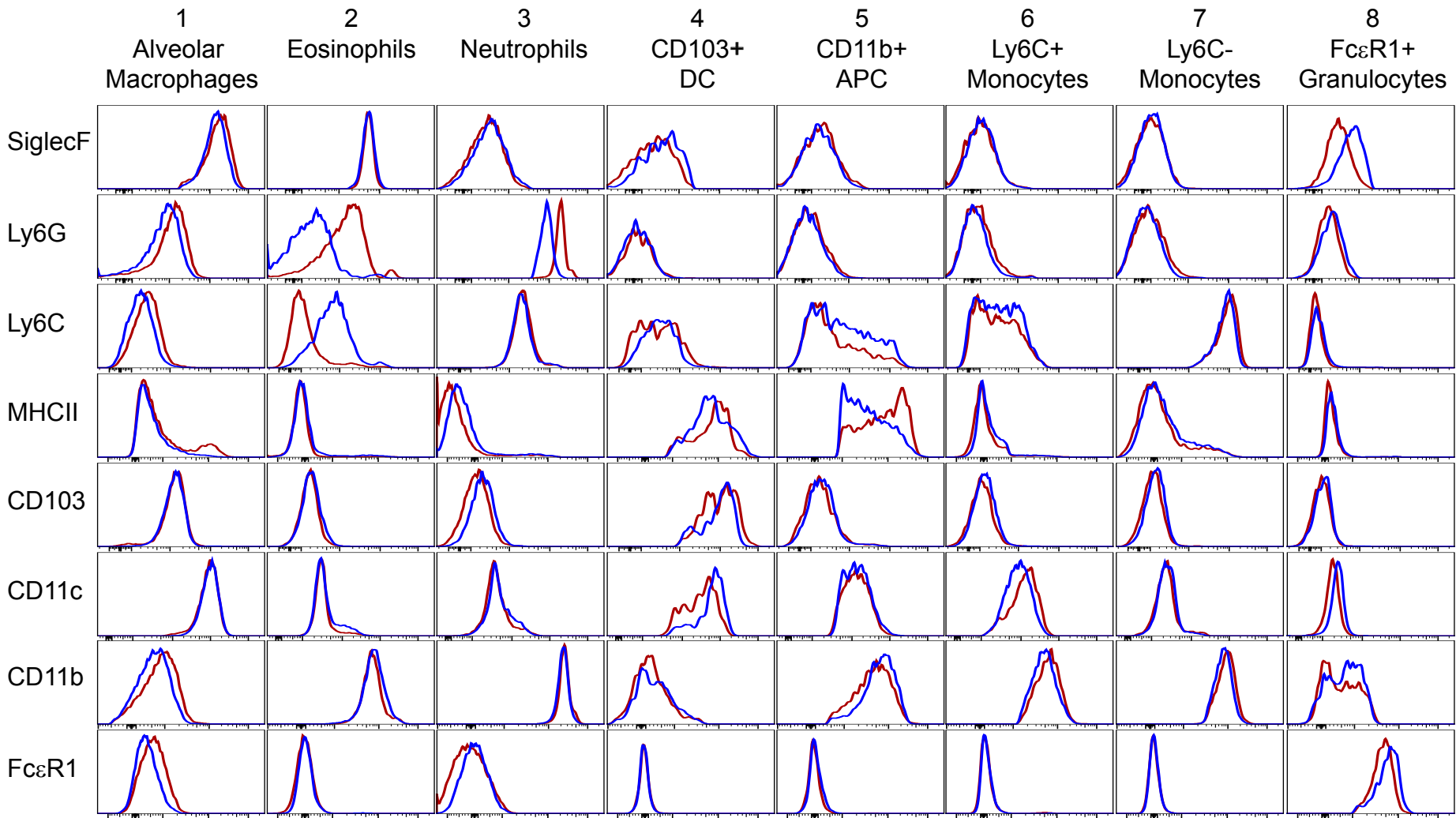

Supplement: Supplementary file 3 [file Image_3.pdf]

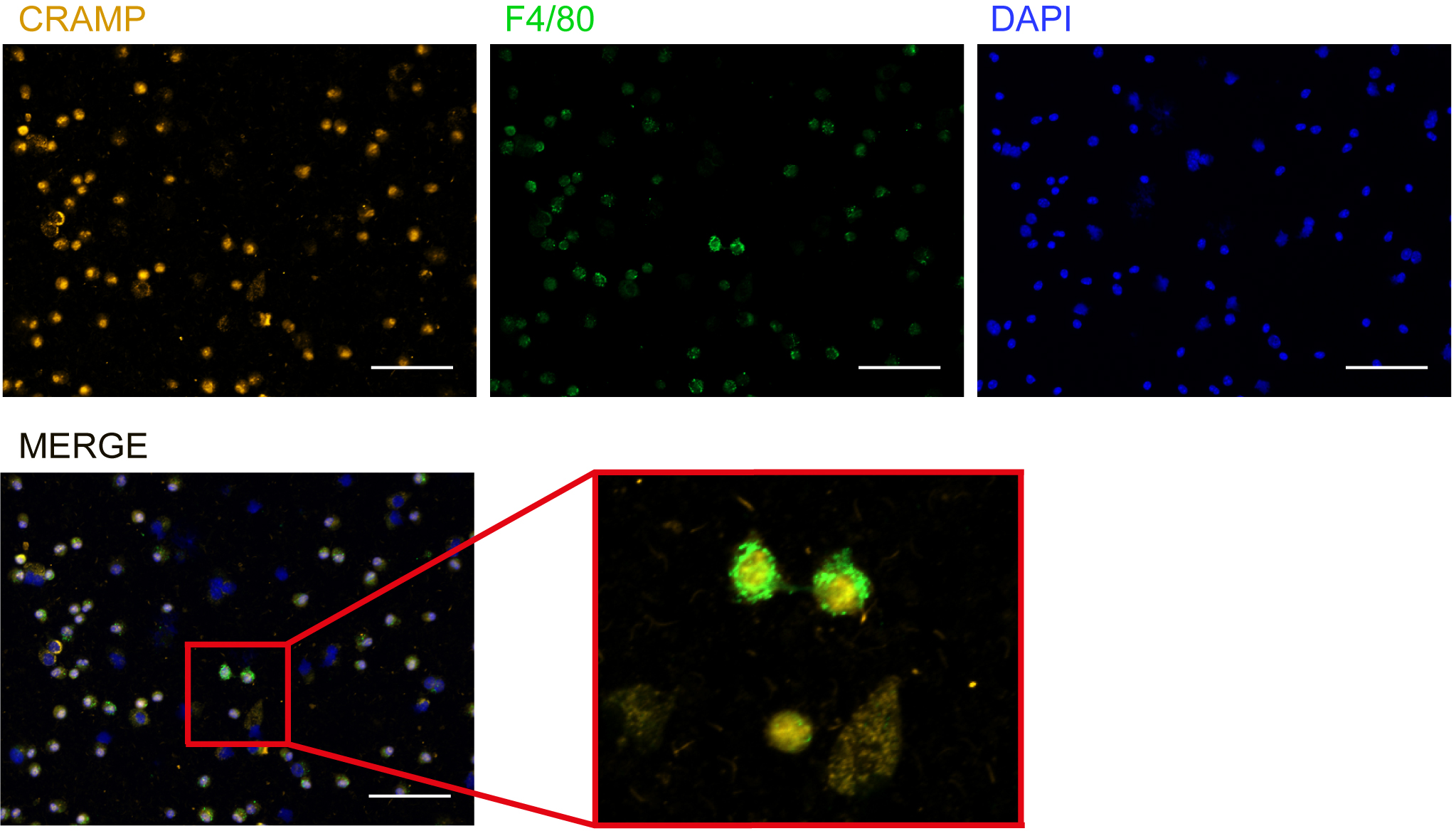

Supplement: Supplementary file 4 [file Image_4.JPEG]
