# Supplementary material for: Early Resistance of Non-virulent Mycobacterial Infection in C57BL/6 Mice Is Associated With Rapid Up-Regulation of Antimicrobial Cathelicidin Camp
Source: Front Immunol. 2018 Sep 3;9:1939. doi: 10.3389/fimmu.2018.01939 (PMC6129578; doi:10.3389/fimmu.2018.01939)
Supplement: Supplementary file 5 [file Data_Sheet_1.DOCX]

***Supplementary Material***

**Early resistance of non-virulent mycobacterial infection in C57BL/6 mice is associated with rapid up-regulation of antimicrobial cathelicidin *Camp***

**Lucille Adam^1*^, Moisés López-González^1^, Albin Björk^2^, Sandra Pålsson^1^, Candice Poux^1^, Marie Wahren-Herlenius^2^, Carmen Fernandez^1#^ and Anna-Lena Spetz^1#*^**

#equal contribution

Correspondence:

Prof. Anna-Lena Spetz, anna-lena.spetz@su.se

Dr. Lucille Adam, lucille.adam@outlook.fr

**Supplementary figure 1: Intranasal BCG infection lead to similar distribution of BCG in the right and left lung lobes.**

BALB/c and C57BL/6 mice were infected with 10^7^ CFU BCG through the intranasal route. Three days post infection the left lung and the right lung lobes were collected, separately disrupted and plated to assess the distribution of the infectious inoculum between both lungs. No significant difference could be highlighted in the BCG count measured post infection between right and left lungs. Mann Whitney test were performed, n=5.

**Supplementary figure 2: Lung cell subsets in the lungs of mice at steady state**

Contour plot representation of the gating strategy used to identify innate immune leukocytes in murine lungs. Lungs were enzymatically and mechanically disrupted and cell suspensions were stained with antibodies described in material and methods before analysis by flow cytometry. Each population described is represented with a red gate and a number. The table depicts the eight different lung cell subsets identified through this gating strategy according to the number associated. The figures correspond to stainings from one representative C57BL/6 mouse analysed at steady state (D0).

**Supplementary figure 3: Comparisons of lung cell subsets in C57BL/6 and BALB/c mice.**

Overlay histograms showing the level of expression of phenotypic markers by the different lung cells subsets in C57BL/6 and BALB/c at steady state.

**Supplementary figure 4: C57BL/6 mice show up-regulation of CRAMP in cells isolated from BAL post-BCG challenge**

Immunofluorescence analyses were performed on cytospun BAL cells. Co-localization of CRAMP and F4/80 in BAL cells of BCG exposed C57BL/6 mice. F4/80 is used as a marker for macrophages (green), CRAMP (orange) and DAPI (blue). Data shown are representative for D1 p.i. (n=6).

**Supplementary Table 1 A:** **Immune related genes regulated one day post BCG inoculation in BALB/c mice.**

List of genes displaying a fold change above 1.5 one day post BCG inoculation with 10^7^ CFU in Balb/c as compared with steady state levels. Data depicts values obtained from 6 number of mice. Green color indicates up-regulated genes and red color indicates down-regulated genes.

| **BALB/c** |  |  |  |  |  |  |  |  |
| --- | --- | --- | --- | --- | --- | --- | --- | --- |
| Gene | Fold change | Log2 fold change | Gene | Fold change | Log2 fold change | Gene | Fold change | Log2 fold change |
| *Cxcl3* | 126.9 | 7.0 | *Ifi204* | 3.4 | 1.8 | *Pdcd1lg2* | 1.8 | 0.9 |
| *Cxcl11* | 36.6 | 5.2 | *Marco* | 3.4 | 1.8 | *Tnfrsf4* | 1.8 | 0.9 |
| *Cxcl10* | 19.2 | 4.3 | *Msr1* | 3.2 | 1.7 | *Fcgr2b* | 1.8 | 0.8 |
| *Cxcl9* | 17.7 | 4.1 | *Tnfrsf8* | 3.1 | 1.6 | *Cd80* | 1.8 | 0.8 |
| *Il1r2* | 15.8 | 4.0 | *Il17a* | 3.1 | 1.6 | *Cybb* | 1.7 | 0.8 |
| *Ccl2* | 14.4 | 3.8 | *Clec5a* | 3.0 | 1.6 | *Cxcr6* | 1.7 | 0.8 |
| *Ccl4* | 13.9 | 3.8 | *Cfi* | 3.0 | 1.6 | *C3* | 1.7 | 0.8 |
| *Cxcl1* | 11.5 | 3.5 | *Cd14* | 2.9 | 1.5 | *Irf7* | 1.7 | 0.8 |
| *Clec4e* | 10.3 | 3.4 | *Pigr* | 2.8 | 1.5 | *Icos* | 1.7 | 0.8 |
| *Tnf* | 9.6 | 3.3 | *H2-Q10* | 2.8 | 1.5 | *Lilra6* | 1.7 | 0.7 |
| *Il6* | 9.2 | 3.2 | *Ccl22* | 2.7 | 1.4 | *Klrc2* | 1.7 | 0.7 |
| *Ccl8* | 8.3 | 3.1 | *Btnl2* | 2.6 | 1.4 | *Ptger4* | 1.6 | 0.7 |
| *Cxcl13* | 7.4 | 2.9 | *Xcl1* | 2.5 | 1.3 | *Foxp3* | 1.6 | 0.7 |
| *Sele* | 7.2 | 2.8 | *Ptafr* | 2.5 | 1.3 | *Csf2* | 1.6 | 0.7 |
| *Ccl3* | 7.0 | 2.8 | *C8g* | 2.4 | 1.3 | *Ncf4* | 1.6 | 0.7 |
| *Itln1* | 5.8 | 2.5 | *Lilrb4* | 2.4 | 1.3 | *Ctla4* | 1.6 | 0.7 |
| *Ltf* | 5.7 | 2.5 | *Trem1* | 2.4 | 1.3 | *Tlr1* | 1.6 | 0.7 |
| *Ccl7* | 5.5 | 2.5 | *Il21r* | 2.3 | 1.2 | *Ccr2* | 1.6 | 0.7 |
| *Il1rn* | 5.4 | 2.4 | *Tnfaip6* | 2.2 | 1.1 | *Fcer1g* | 1.6 | 0.7 |
| *Ccl19* | 5.4 | 2.4 | *Fcer1a* | 2.1 | 1.1 | *Ccl6* | 1.6 | 0.7 |
| *Ccl20* | 5.3 | 2.4 | *S100a9* | 2.1 | 1.1 | *Irf3* | 1.6 | 0.7 |
| *Pdcd1* | 4.8 | 2.3 | *Il17rb* | 2.1 | 1.1 | *Ifitm1* | 1.6 | 0.6 |
| *Ccl12* | 4.3 | 2.1 | *Tnfsf11* | 2.1 | 1.0 | *Cd40lg* | 1.6 | 0.6 |
| *Tigit* | 4.1 | 2.0 | *Cxcr1* | 2.0 | 1.0 | *Ccl24* | 1.6 | 0.6 |
| *Il23r* | 4.0 | 2.0 | *Cxcr2* | 2.0 | 1.0 | *Ikbke* | 1.5 | 0.6 |
| *Il9* | 3.8 | 1.9 | *Fcgr1* | 2.0 | 1.0 | *Tlr2* | 1.5 | 0.6 |
| *Il17f* | 3.7 | 1.9 | *Ccr5* | 2.0 | 1.0 | *Lair1* | 1.5 | 0.6 |
| *Cfb* | 3.7 | 1.9 | *Itgam* | 2.0 | 1.0 | *Cd96* | 1.5 | 0.6 |
| *Il12b* | 3.5 | 1.8 | *Cfp* | 1.9 | 0.9 | *Fcgr4* | 1.5 | 0.6 |
| *Il1b* | 3.5 | 1.8 | *S100a8* | 1.9 | 0.9 | *Tgfbi* | 1.5 | 0.6 |
| *Ccl9* | 3.5 | 1.8 | *Pla2g2e* | 1.9 | 0.9 | *Gp1bb* | 2.0 | 1 |
| *Tnfrsf9* | 3.4 | 1.8 | *Csf3r* | 1.9 | 0.9 | *Ppbp* | 1.6 | 0.7 |

**Supplementary Table 1 B: Immune related genes regulated one day post BCG inoculation in C57BL/6 mice.**

List of genes displaying a fold change above 1.5 one day post BCG inoculation with 10^7^ CFU in C57BL/6 as compared with steady state levels. Data depicts values obtained from 6 number of mice. Green color indicates up-regulated genes and red color indicates down-regulated genes.

| **C57BL/6** | |  |  |  |  |
| --- | --- | --- | --- | --- | --- |
| Gene | Fold change | Log2 fold change | Gene | Fold change | Log2 fold change |
| *Cxcl3* | 8.3 | 3.1 | *Il23r* | 3.6 | 1.9 |
| *Itln1* | 2.7 | 1.4 | *Tigit* | 2.6 | 1.4 |
| *Camp* | 2.5 | 1.3 | *Il12rb1* | 2.2 | 1.2 |
| *Ltb4r2* | 2.2 | 1.1 | *Slamf1* | 2.2 | 1.1 |
| *Gpr44* | 2.1 | 1.0 | *Cd69* | 2.2 | 1.1 |
| *Tnf* | 1.9 | 0.9 | *Tnfrsf8* | 2.0 | 1.0 |
| *Tnfrsf9* | 1.8 | 0.9 | *Klra4* | 1.9 | 0.9 |
| *Ccl20* | 1.8 | 0.8 | *Cd46* | 1.8 | 0.8 |
| *Batf3* | 1.8 | 0.8 | *Cd96* | 1.8 | 0.8 |
| *Mapk11* | 1.8 | 0.8 | *Il1b* | 1.7 | 0.8 |
| *Cd79a* | 1.7 | 0.8 | *Ltf* | 1.7 | 0.8 |
| *Clec4e* | 1.6 | 0.7 | *Tnfrsf13c* | 1.7 | 0.8 |
| *Cxcl9* | 1.6 | 0.7 | *Lcp2* | 1.7 | 0.7 |
| *Pdcd1* | 1.6 | 0.7 | *Il17rb* | 1.6 | 0.7 |
| *Folr4* | 1.5 | 0.6 | *Nfil3* | 1.6 | 0.7 |
| *Cd28* | 1.5 | 0.6 | *Cxcl13* | 1.6 | 0.7 |
| *Il11ra1* | 1.5 | 0.6 | *Ptgs2* | 1.6 | 0.7 |
| *Ccl4* | 1.5 | 0.6 | *Il28a* | 1.6 | 0.7 |
| *Il1r2* | 1.5 | 0.6 | *Gm10499* | 1.6 | 0.6 |
|  |  |  | *Cfi* | 1.6 | 0.6 |
|  |  |  | *C8g* | 1.6 | 0.6 |
|  |  |  | *Icam5* | 1.5 | 0.6 |
